# Revisiting the interaction between complement lectin pathway protease MASP-2 and SARS-CoV-2 nucleoprotein

**DOI:** 10.3389/fimmu.2024.1419165

**Published:** 2024-06-07

**Authors:** Isabelle Bally, Guillaume Drumont, Véronique Rossi, Serafima Guseva, Maiia Botova, Jean-Baptiste Reiser, Michel Thépaut, Sebastian Dergan Dylon, Chantal Dumestre-Pérard, Christine Gaboriaud, Franck Fieschi, Martin Blackledge, Pascal Poignard, Nicole M. Thielens

**Affiliations:** ^1^ Univ. Grenoble Alpes, CEA, CNRS, IBS, Grenoble, France; ^2^ Laboratory of Immunology, Grenoble Alpes University Hospital, Grenoble, France; ^3^ Laboratory of Virology, Grenoble Alpes University Hospital, Grenoble, France

**Keywords:** complement system, lectin pathway, MASP-2, SARS-CoV-2, nucleoprotein

## Abstract

Complement activation is considered to contribute to the pathogenesis of severe SARS-CoV-2 infection, mainly by generating potent immune effector mechanisms including a strong inflammatory response. Involvement of the lectin complement pathway, a major actor of the innate immune anti-viral defense, has been reported previously. It is initiated by recognition of the viral surface Spike glycoprotein by mannose-binding lectin (MBL), which induces activation of the MBL-associated protease MASP-2 and triggers the proteolytic complement cascade. A role for the viral nucleoprotein (N) has also been reported, through binding to MASP-2, leading to protease overactivation and potentiation of the lectin pathway. In the present study, we reinvestigated the interactions of the SARS-CoV-2 N protein, produced either in bacteria or secreted by mammalian cells, with full-length MASP-2 or its catalytic domain, in either active or proenzyme form. We could not confirm the interaction of the N protein with the catalytic domain of MASP-2 but observed N protein binding to proenzyme MASP-2. We did not find a role of the N protein in MBL-mediated activation of the lectin pathway. Finally, we showed that incubation of the N protein with MASP-2 results in proteolysis of the viral protein, an observation that requires further investigation to understand a potential functional significance in infected patients.

## 1 Introduction

As is well known, SARS-CoV-2, the human coronavirus associated with severe acute respiratory syndrome (SARS), is responsible for the COVID-19 pandemic. Evidence for involvement of an excessive inflammatory immune response in the disease severity has emerged (1). The complement system is a major actor of anti-microbial host innate immune defense. Complement activation orchestrates immune and inflammatory processes aiming at pathogen elimination but that might contribute to excessive inflammation and tissue injury if not properly regulated. Clinical studies have provided evidences for strong complement activation in the serum and lungs of critically ill COVID-19 patients (2–4) and a few case reports have shown a beneficial effect of blocking complement at the levels of C3 or C5 components, which are involved in the generation of the pro-inflammatory C3a and C5a mediators (5–7).

Complement can be activated through three pathways, classical, lectin and alternative, which involve different recognition proteins but converge at the level of C3 complement component cleavage. The lectin complement pathway is of particular importance in the context of viral infection. It is initiated by recognition of viral surface glycoproteins by soluble oligomeric proteins of the collectin family, including mannose-binding lectin (MBL) and ficolins, which associate with MBL-associated serine proteases (MASPs) to trigger the complement cascade. In line with clinical studies reporting that MBL likely contributes to complement activation and uncontrolled inflammation (8–11), a few case reports showed successful treatment of COVID-19 patients with Narsoplimab, a human monoclonal antibody against MASP-2, the effector protease of the lectin pathway, known to inhibit complement activation and with anticoagulant effects (12–14). Inhibition of the lectin pathway activation by a monoclonal antibody targeting MASP-2 has also been shown to reduce acute respiratory distress syndrome (ARDS) severity in mouse models of SARS-CoV-2 infection (15, 16).

The molecular determinants of the lectin pathway activation have been previously investigated and MBL has been shown to interact with SARS-CoV-2 Spike surface glycoprotein and to trigger complement activation (13, 17). In addition, *in vitro* studies have provided evidence for blockage of viral entry by MBL, suggesting a protective function (17, 18). A role for the SARS-CoV-2 N protein (alias nucleoprotein or nucleocapsid) has also been reported through interaction with the catalytic domain of MASP-2, leading to MBL-dependent overactivation of this protease and potentiation of the lectin pathway (13, 15). However, the participation of the N protein in activation of the lectin pathway has been questioned in a recent conference presentation abstract (19).

The aim of the present study was to reinvestigate the molecular interactions of the SARS-CoV-2 N protein with the MASP-2 protease of the lectin pathway and its possible effect on complement activation. We took advantage of our capacity to express recombinant MASP-2 as a full-length protease, in addition to its catalytic domain, and used two forms of recombinant viral N protein, either expressed in bacteria or secreted by mammalian cells. Using *in vitro* settings, we could not confirm the interaction of the viral N protein with the catalytic domain of MASP-2 and a role of N protein in activation of the lectin complement pathway. We also identified N protein as a substrate of the MASP-2 protease, which, to our knowledge, had not been reported before.

## 2 Materials and methods

### 2.1 Proteins

#### 2.1.1 Recombinant MASP-2

Recombinant human MASP-2 with a C-terminal Flag tag was produced by stable transfection of 293-F cells using a pcDNA3.1_MASP-2-Flag expression vector as described by Lorvellec et al. (20). The expression plasmid coding for recombinant MASP-2 stabilized in a proenzyme form by mutation of the active site Ser 618 into Ala (mature protein numbering) was generated with the QuickChange II XL site-directed mutagenesis kit (Agilent Technologies), using the mutagenic primers described by Zundel et al. (21) and the pcDNA3.1_MASP-2-Flag plasmid as template. Each plasmid was used for stable transfection of Freestyle 293-F cells (ThermoFisher Scientific) and the recombinant proteins were purified from cell culture supernatants by anti-FLAG and C1q affinity chromatography, as described by Lorvellec et al. (20). MASP-2 molar concentration was estimated using a M_r_ value of 75,100 and an absorbance (0.1%, 1 cm) at 280 nm (A_0.1%, 1 cm_) of 1.57. SDS- PAGE analysis of purified MASP-2 showed that it was recovered in an active form, as assessed by the presence of the A and B chains under reducing conditions (**Supplementary Figure S1A**), whereas the S618A mutant exhibited a single band corresponding to the proenzyme form of the protease.

The expression plasmid coding for the catalytic domain of MASP-2 encompassing the CCP1, CCP2 and serine protease (SP) domains (amino acids 282–671 of mature protein, named CCP1,2-SP) was generated from the pcDNA3.1_MASP-2-Flag plasmid by deletion of the N-terminal CUB1-EGF-CUB2 sequence using the QuickChange II XL site-directed mutagenesis kit. This plasmid served as a template to generate the expression plasmid coding for the S618A mutant of MASP-2 catalytic domain. Both catalytic domains were purified from cell culture supernatant on an anti-FLAG M2 agarose column (Sigma-Aldrich) as described by Lorvellec et al. (20). Most of the recombinant proteins were recovered in the flow through, dialyzed against 20 mM Na_2_HPO_4_, 5 mM EDTA, pH 8.6 and loaded onto a Q-Sepharose Fast Flow column (Cytiva) equilibrated in the same buffer. Elution was carried out by applying a linear gradient from 0 to 350 mM NaCl in the same buffer, as described previously (22). Fractions containing the recombinant proteins were identified by Western blot analysis, dialyzed against Tris-buffered saline, pH 7.5 (TBS, EuroMedex) and concentrated by ultrafiltration up to 0.34 mg/ml. The concentration of MASP-2 catalytic domain was estimated using M_r_ and A_0.1%, 1 cm_ values of 43,540 and 1.84, respectively. As observed for full-length MASP-2, the catalytic domain of the protease was produced in an activated form whereas its S618A mutant was stabilized in the proenzyme form (**Supplementary Figure S1A**). The purity of the fragment was estimated to be >85%.

#### 2.1.2 Other human complement proteins

Activated C1r and C1s proteases were purified from human serum as described by Arlaud et al. (23), and their concentration estimated using M_r_ and A_0.1%, 1 cm_ of 86,300 and 1.24 (C1r), and 78,900 and 1.45 (C1s).

Recombinant mannose-binding lectin (MBL) was produced in 293-F cells and purified by affinity chromatography on N-acetylglucosamine-agarose as described previously (24). Its molar concentration was estimated using A_0.1%, 1 cm_ = 0.78 and M_r_ = 305,000, assuming a majority of tetrameric species present in the samples.

#### 2.1.3 Recombinant SARS-CoV-2 N and S proteins

Two recombinant forms of N protein were used, expressed either in bacteria (N-Bact) or in mammalian Expi293 cells (N-Expi). N-Bact (aa 1–419 with an N-terminal His6-tag) was expressed in *Escherichia coli* BL21 (DE3) and purified by Ni-affinity and size exclusion chromatographies as described by Bessa et al. (25). N-Bact concentration was estimated using a M_r_ of 44,000 and an A_0.1%, 1 cm_ of 0.95. SDS-PAGE analysis showed a major band at 55 kDa and a minor degradation product (**Supplementary Figure S1B**). A pcDNA3.4 plasmid encoding N-Expi (aa 1–419 with mouse IgG2 Heavy Chain signal peptide and a C-terminal His6-tag) was kindly provided by Dr S. Simon, CEA- I2BC, Saclay. The recombinant protein was produced using the Expi293 transient expression system as described by the manufacturer (ThermoFisher Scientific). The cell culture supernatant was loaded using recommended procedure on a Hitrap Chelating HP column (Cytiva) equilibrated in TBS containing 5 mM imidazole and the recombinant N protein was eluted using a 5–500 mM imidazole gradient in TBS. The protein was dialyzed against TBS and concentrated to 0.36 mg/ml. N protein concentration was estimated using M_r_ = 47,000 and A_0.1%, 1 cm_ = 0.95. N-Expi analysis by SDS-PAGE showed a large band at around 60 kDa likely reflecting the heterogeneous glycosylated state of the protein, with some degradation products (**Supplementary Figure S1C**), as reported previously for a commercial protein produced with a proprietary signal peptide sequence (26).

The trimeric ectodomain of prefusion stabilized S protein was expressed in Expi293F cells and purified by Ni-affinity and size exclusion chromatography as described by Thépaut et al. (27). S protein was quantified using M_r_ = 420,000 and A_0.1%, 1 cm_ = 1.04.

### 2.2 Characterization of N interaction with complement proteins by surface plasmon resonance (SPR)

SPR interaction analyses were performed at 25°C on a T200 instrument (Cytiva). N proteins were captured through their His-tag using a penta-His antibody (Qiagen) immobilized on a CM5 Series S sensorchip (Cytiva). The antibody was diluted to 20 µg/ml in 10 mM sodium acetate pH 4.5 and 6,300–7,300 RU were covalently coupled to two adjacent flow cells using the amine coupling chemistry in HBS-EP+ (Cytiva). N proteins (1,200–2,300 RU) were captured on the Penta-His antibody by injecting N-Bact (4 µg/ml) or N-Expi (18 µg/ml) in TBS buffer, pH 7.4, Tween 20 0.05% (TBS-T). Binding was measured at a flow rate of 20 µl/min in TBS-T containing 2 mM CaCl_2_ (TBS-Ca-T). The specific binding signal was obtained by subtracting the signal over the reference surface (flow cell with immobilized Penta-His antibody and without N captured). Regeneration of the surfaces was achieved by 10 µl injections of 10 mM glycine pH 2.

Kinetic data were recorded in single cycle kinetics mode, using five concentrations of MASP-2 S618A (ie 37.5, 75, 150, 300 and 600 nM). Buffer blanks were subtracted from the data sets (double referencing). Global fitting of the data to the 1:1 Langmuir binding model was performed using the Biacore T200 evaluation 3.2 software (Cytiva). The apparent equilibrium dissociation constants (*K*_D_) were calculated from the ratio of the dissociation and association rate constants (*k*_d_/*k*_a_). Chi^2^ values were below 1 in all cases. Kinetic values represent means ± SD (n = 2 or 3, as indicated).

### 2.3 N proteolysis by MASP-2 and homologous proteases

Recombinant SARS-CoV-2 N and S proteins were incubated with complement serine proteases using an enzyme/substrate molar ratio of 1/10 in TBS for 90–120 min at 37°C. The samples were transferred to ice and immediately reduced with 50 mM dithiothreitol in 0.1 M Tris-HCl, pH 8.0 containing 4 M urea and 1% SDS for 60 min at 37°C and alkylated with 140 mM iodoacetamide for 20 min at 37°C. Samples were analyzed by SDS-PAGE and staining with Instant Blue dye reagent (Expedeon) or Quick Coomassie Stain (Clinisciences).

### 2.4 Activation of the lectin complement pathway

Complement activation via the MBL-dependent lectin pathway was investigated using a C4b deposition ELISA, as described by Lacroix et al. (28). S, N and control mannan (at concentrations of 10, 20 and 50 µg/ml, respectively, in PBS) were coated on microplates and incubated with either 10% human MBL-deficient human serum (Statens Serum Institute, Copenhagen, Denmark) reconstituted with 5 μg/ml recombinant human MBL, or 4% normal human serum (NHS). NHS was obtained from the Etablissement Français du Sang Rhône-Alpes (agreement number EFS AURA 21–001 regarding its use in research). The potential contribution of viral N protein to mannan- or S protein-mediated complement activation was assayed by adding soluble N protein (10 µg/ml) in the serum.

### 2.5 Statistical analysis

Where applicable, Prism GraphPad software was used for the statistical analyses of data. Comparisons among groups were performed using one or two-way ANOVA followed by Bonferroni’s or Tukey’s multiple comparison tests.

## 3 Results

Contradictory results have been reported about the interaction of the N protein of SARS-CoV-2 and complement proteins and its consequences, more particular its capacity to enhance activation of the lectin complement pathway through interaction with the MASP-2 protease (15). Published experiments have been performed using different sources of recombinant N (commercial or home-made), including a fusion protein with a signal peptide, secreted as a glycosylated protein by eukaryotic cells, or a non-glycosylated form produced in eukaryotic cells without signal peptide (26) or by bacteria. We produced both forms (N-Expi and N-Bact) to reinvestigate the capacity of N protein to interact with the MASP-2 protease of the lectin complement pathway. The full-length protease was expressed in 293-F cells, either as a wild-type active protease or stabilized in a proenzyme form by mutation of the active site Ser by Ala, and its catalytic domain (active or proenzyme) was also produced using the same cells (**Supplementary Figure S1**).

### 3.1 N protein interaction with MASP-2

**Figure 1:**
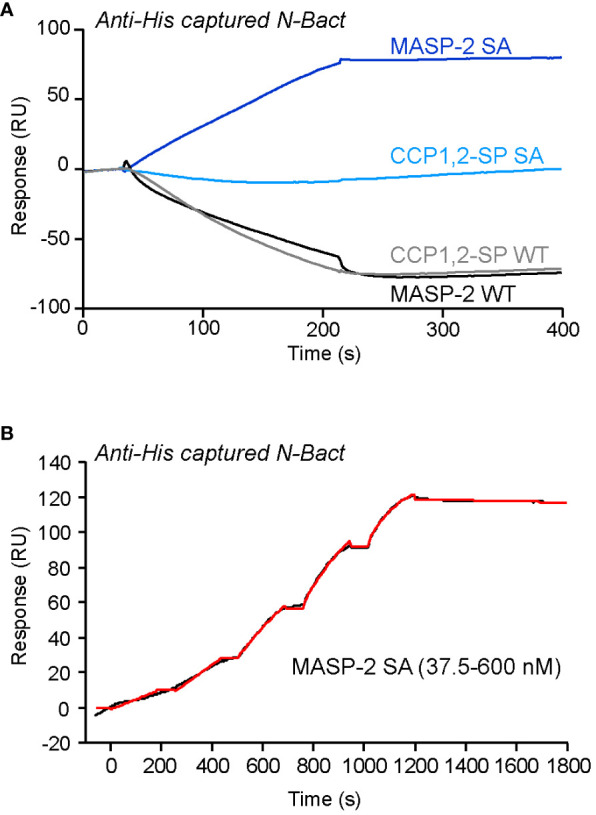
SPR analyses of the interaction of MASP-2 proteins with N-Bact. **(A)** MASP-2 proteins (250 nM) were injected over 2,350 RU of N-Bact captured by covalently immobilized Penta-His antibody in TBS-Ca-T, pH 7.4 at a flow rate of 20 µl/min. The specific binding signal was obtained by subtracting the signal over the reference surface (flow cell with immobilized Penta-His antibody and without N captured). **(B)** MASP-2 S618A was serially diluted and injected at five increasing concentrations in single cycle kinetics mode over captured N-Bact (2,450 RU) in TBS-Ca-T at a flow rate of 20 μl/min. The fit (shown by a red line) was obtained by global fitting of the data to a Langmuir 1:1 binding model. The data shown are representative of three separate experiments on different surfaces.

We investigated the interaction of N with MASP-2 by SPR spectroscopy, using oriented capture of both N protein forms through their His-tag and injection of the soluble protease. As illustrated for N-Bact in **Figure 1A**, injection of proenzyme MASP-2 (S618A mutant) resulted in a signal increase, reflecting binding of the protease, whereas no significant binding was detected for the proenzyme form of MASP-2 catalytic domain. In contrast, a decrease of the signal was observed reproducibly when injecting the wild-type forms of both MASP-2 and its catalytic domain (**Figure 1A**). Comparable results were obtained using recombinant N-Expi protein, except that a weak binding of wild-type MASP-2 was observed (**Supplementary Figure S2A**). Kinetic analysis of proenzyme MASP-2 binding to captured N-Bact (**Figure 1B**) yielded an association rate constant (*k*_a_) of 1.39 ± 0.35 x 10^4^ M^-1^ s^-1^, a dissociation rate constant (*k*_d_) of 1.50 ± 0.83 x 10^-4^ s^-1^, with a resulting apparent equilibrium dissociation constant (*K*_D_) of 12.3 ± 9.3 nM (n = 3), reflecting high affinity. Similar values were obtained using captured N-Expi (**Supplementary Figure S2B**), with *k*_a_ = 1.44 ± 0.63 x 10^4^ M^-1^ s^-1^, *k*_d_ = 1.72 ± 0.29 x 10^-4^ s^-1^ and *K*_D_ = 13.7 ± 4.0 nM (n = 2). The observed decrease of the signal when injecting the active proteases was intriguing and led us to hypothesize that N protein might be cleaved by MASP-2, which would result in loss of captured ligand over time.

### 3.2 N protein cleavage by MASP-2

**Figure 2:**
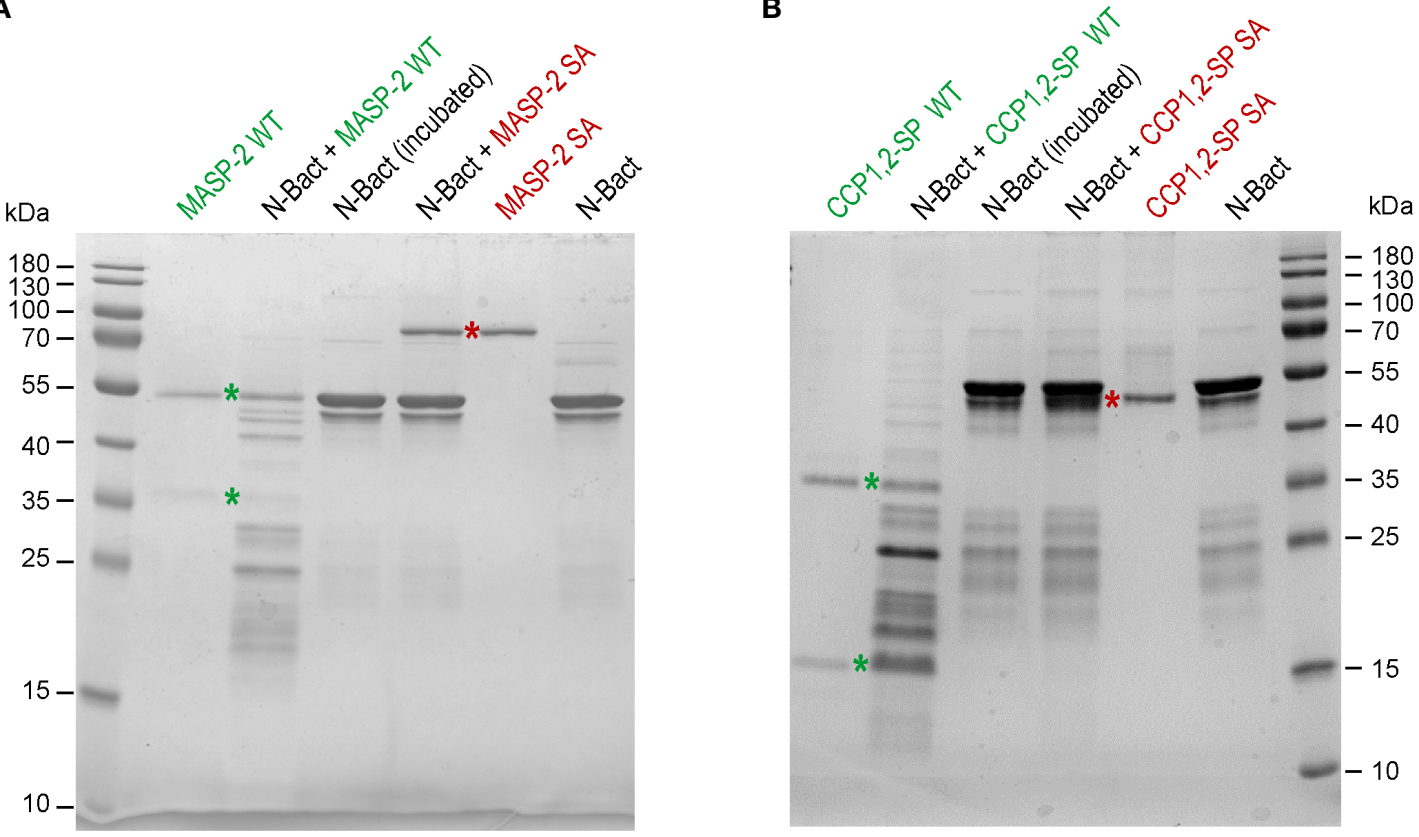
MASP-2 proteolytic activity on N-Bact protein. N-Bact was incubated with wild-type activated MASP-2 **(A)** or its catalytic CCP1,2-SP fragment **(B)**, their proenzyme S618A counterparts or TBS for 90 min at 37°C. The digestion products and the control proteases and substrate were loaded on 12.5% acrylamide gels and SDS-PAGE analysis was performed under reducing conditions. The bands corresponding to the two chains of activated wild-type MASP-2 or its catalytic domain and to their proenzyme counterparts are indicated by green and red stars, respectively. The molecular masses (kDa) of the markers are indicated. Each gel shown is representative from three experiments.

**Figure 3:**
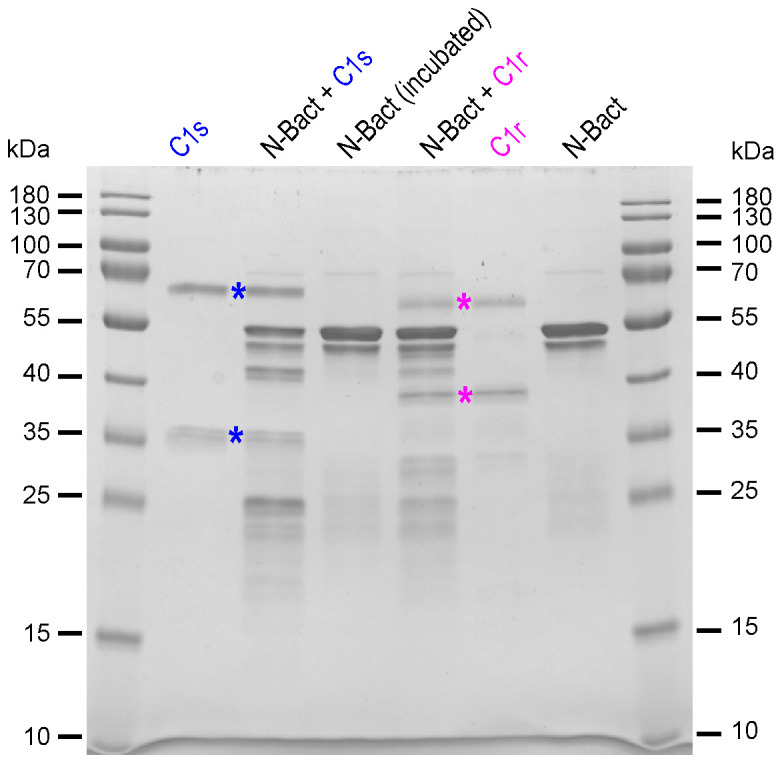
Proteolytic activity of complement C1s and C1r proteases on N-Bact protein. N-Bact was incubated with activated C1s, activated C1r or TBS for 90 min at 37°C. The digestion products and the control proteases and substrate were loaded on a 12.5% acrylamide gel and SDS-PAGE analysis was performed under reducing conditions. The bands corresponding to the two chains of activated C1s and C1r are indicated by blue and pink stars, respectively. The molecular masses (kDa) of the marker are indicated. A gel representative from two experiments is shown.

In order to check the above hypothesis, N proteins were incubated with full-length MASP-2, either activated (wild-type) or stabilized in proenzyme form (S618A mutant). Incubation of N-Bact with 10% active MASP-2 (molar ratio) for 90 min at 37°C resulted in disappearance of the band corresponding to the intact N protein with concomitant appearance of several degradation fragments of lower molecular weights (**Figure 2A**). Incubation of N-Bact with proenzyme MASP-2 or buffer did not induce any change, indicating that the observed proteolysis was due to the enzymatic activity of MASP-2. Incubation with the catalytic domain of MASP-2, either activated or stabilized in a proenzyme form, under the same molar ratio and reaction conditions, resulted in similar patterns (**Figure 2B**), thus confirming the capacity of MASP-2 enzyme to cleave N protein. Proteolysis of N-Expi by the wild-type MASP-2 proteases could also be observed (**Supplementary Figure S3A**), although the pattern was less clear due to the glycosylated and partially proteolysed state of the purified protein. We also investigated MASP-2 capacity to cleave the Spike protein of SARS-CoV-2. As shown in **Supplementary Figure S3B**, no detectable cleavage of S was observed following incubation for 2 h at 37°C with 10% enzyme: substrate (recombinant trimeric Spike ectodomain) ratio. The activity of MASP-2 was further compared with that of the two homologous proteases of the classical complement pathway, C1r and C1s. C1s has a substrate specificity similar to MASP-2 by cleaving complement proteins C4 and C2 whereas C1r only activates zymogen C1s. As shown in **Figure 3**, incubation with C1s resulted in very partial proteolysis of N-Bact protein, characterized by generation of fragments similar to those observed in the case of MASP-2. A similar profile was observed with C1r, although the cleavage was even less efficient than with C1s. These results indicate that complement proteases C1s and C1r are much less efficient than MASP-2 in cleavage of N protein.

### 3.3 N protein and activation of the lectin complement pathway

**Figure 4:**
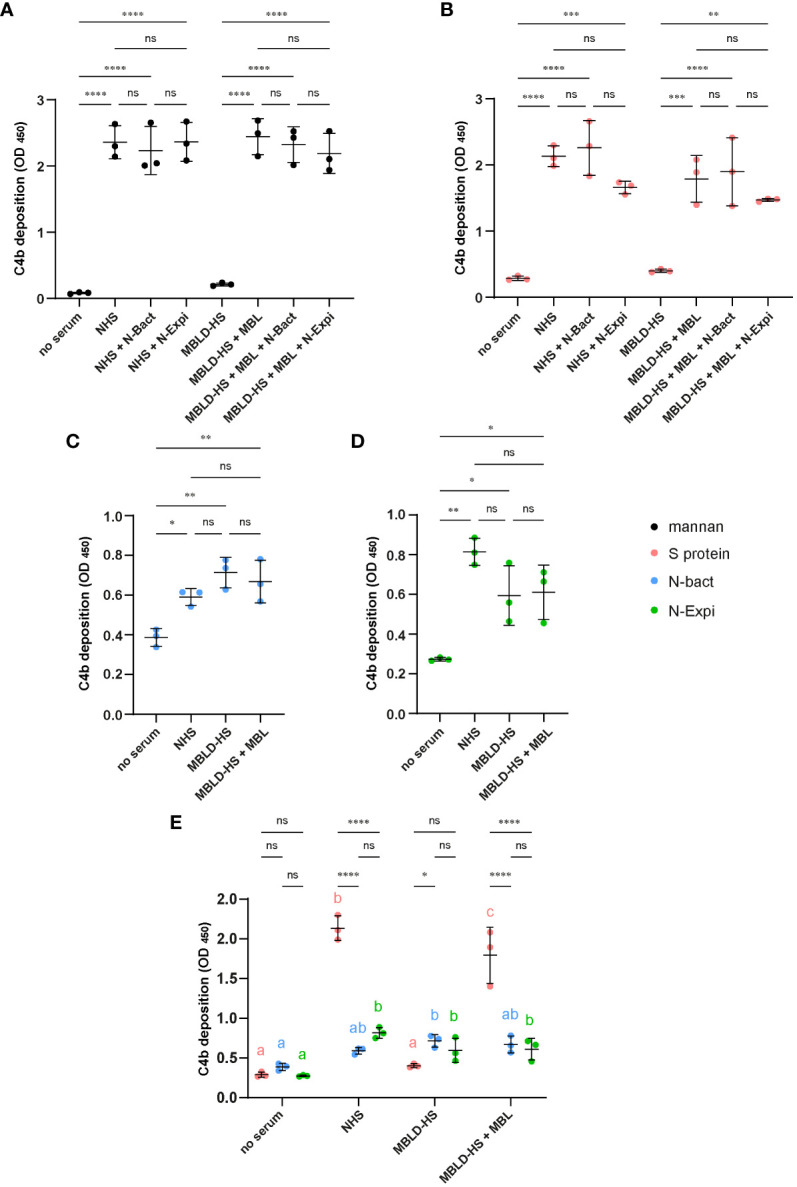
**(A, B)** Effect of N proteins on mannan- and S protein-mediated complement activation. Normal human serum (NHS) or MBL-deficient serum (MBLD-HS) reconstituted with MBL were added to microwells coated with 50 µg/ml mannan **(A)** or 10 µg/ml S protein **(B)** in the presence or absence of 10 µg/ml N-Bact or N-Expi. Incubation without serum or with MBLD-HS served as controls. Complement activation was measured by a C4b deposition ELISA. **(C, D)** N protein-mediated complement activation. Normal human serum (NHS) or MBL-deficient serum (MBLD-HS) reconstituted with MBL were added to microwells coated with 20 µg/ml N-Bact **(C)** or N-Expi **(D)** and complement activation was measured as in **(A, B)**. All data represent mean and SD of three independent experiments performed in duplicate. Statistical analysis was performed by one-way ANOVA followed by Bonferroni’s **(A, B)** or Tukey’s **(C, D)** multiple comparisons. **(E)** Comparison of the data obtained with the different sera and coated S protein **(B)**, N-Bact **(C)** and N-Expi **(D)**. Statistical analysis was performed by two-way ANOVA, followed by Tukey’s multiple comparison test. Simple effects within sera are presented using compact letter display. Different letters with the same color indicate statistically significant differences (p<0.05). *p<0.05; **p<0.01; ***p<0.001; ****p<0.0001; ns, no statistically significant differences.

It has been demonstrated that SARS-CoV-2 Spike protein is able to activate the lectin complement pathway (13, 15, 17) but the role of the viral N protein is controversial (13, 19). Activation of the lectin complement pathway was measured by C4b deposition in wells coated with either mannan or the viral S protein, following incubation with either MBL-deficient HS reconstituted with MBL or normal HS (NHS). As expected, control mannan and S protein triggered MBL-dependent activation of the lectin complement pathway (**Figures 4A, B**). Addition of either N-Bact or N-Expi to the sera (NHS or MBL-deficient HS reconstituted with MBL) did not induce statistically significant differences in the level of C4b deposition (**Figures 4A, B**). The capacity of the N protein to trigger complement activation complement was also assayed directly by coating N-Bact or N-Expi. We observed weak, although significant C4b deposition using both sera (NHS and MBL-deficient HS), with no effect of reconstitution of the MBL-deficient serum with MBL (**Figures 4C, D**). This result indicates that C4b deposition on coated N proteins was clearly not the result of MBL-dependent activation of the lectin pathway. Comparison between C4b deposition induced by SARS-CoV-2 S and both N protein forms confirmed that the Spike protein was much more efficient in triggering activation of the lectin complement pathway (**Figure 4E**).

## 4 Discussion

In the present study, we revisited the complement activating capacity of the N protein and the molecular interactions between the MASP-2 protease of the lectin complement pathway and the nucleoprotein of SARS-CoV-2, using home-made and well characterized recombinant SARS-CoV-2 N and human complement MASP-2 proteins. It seemed important to use the N protein either produced in bacteria or secreted by mammalian cells since differences have been reported between both proteins, mainly because the secreted protein contains numerous post-translational modifications, including N- and O-glycosylations (26). Contradictory results have been reported regarding the recombinant N protein produced in HEK293 cells without signal sequence, which was characterized as a non-glycosylated (26) or glycosylated protein (29). Our assumption of the relevance of the bacterial recombinant N protein is based on the fact that the native viral N protein is an intracellular protein lacking a signal sequence, which is expected to prevent some post-translational modifications such as N-glycosylation. In addition, using the recombinant protein either produced in *E. coli* or secreted by CHO cells, Rump et al. have demonstrated that glycosylation of the N-protein masks some of its immunodominant antibody epitopes (30).

Several studies have demonstrated that interaction of the Spike glycoprotein of SARS-coronaviruses with MBL triggers activation of the lectin complement pathway (13, 17, 18), which was confirmed in the present study. The N protein was proposed to potentiate lectin pathway activation (15) or to directly trigger complement activation (13), but we could not detect an effect of soluble N protein, produced either in bacteria or in Expi293 cells, on mannan- or SARS-CoV-2 Spike protein-mediated complement activation. Although weak C4b deposition was observed on coated N proteins, it did not arise from MBL-dependent activation of the lectin complement pathway. Interestingly, this observation is in accordance with data reported recently by Kocsis et al. in a conference presentation abstract (19), who concluded that the N protein activates the alternative complement pathway but not the lectin or classical pathways. The fact that we detected weak C4b deposition on coated N proteins likely reflects some activation of the classical pathway, which might be caused by the presence of anti-N protein antibodies. Indeed our NHS was collected after the pandemic and, given the known high immunogenicity of N protein, the presence of antibodies is highly plausible. It should be mentioned that our data are not in contradiction with the reported therapeutic benefice of MASP-2 specific inhibition by Narsoplimab. Indeed, since this protease is essential to trigger the lectin complement pathway, its inhibition will anyway reduce complement activation and its potential noxious consequences.

A new result of the present study resides in the observed capacity of the MASP-2 protease to cleave the N protein, which was assessed by comparing proenzyme and activated forms of the full-length protease and its catalytic domain. This cleavage property implies transient interaction between the active protease and its substrate, which could not be detected in our SPR measurements due to the proteolysis of N protein. However, we observed high affinity interaction of the viral protein (either produced in bacteria or secreted by Expi293 cells) with full-length MASP-2 stabilized in the proenzyme form. The *K*_D_ values determined by SPR were in the 10 nM range, lower than those determined by Gao et al. (15) for SARS-CoV-1 and SARS-CoV-2 N proteins (268 and 514 nM, respectively). However, direct comparison is not possible due to the lack of experimental details of the latter study, regarding the nature of immobilized ligands and soluble analytes, the protease activation state and the immobilization procedure. In the case of N-Expi, a weak interaction was observed with wild-type MASP-2, which might explain previously reported N protein-MASP-2 interactions (15). The possible biological significance of the interaction between proenzyme MASP-2 and the N-protein is difficult to predict from *in vitro* experiments using isolated recombinant proteins. The concentration of MASP-2 in serum is very low (0.5 µg/ml) (31) which corresponds to a molar concentration of 6 nM, lower than the determined *K*_D_ value for the interaction with the N protein (between 10 and 20 nM). In addition, all proenzyme MASP-2 in serum forms complexes with multiple complement pattern recognition proteins such as MBL, ficolins and collectin-11 (31–33), which likely restricts its interaction capacities.

In contrast to the two studies reporting interaction of the catalytic domain of MASP-2 with the N proteins of the SARS-CoV-2, SARS-CoV and MERS-CoV (13, 15), we could not detect any interaction of SARS-CoV-2 N protein with the catalytic domain of MASP-2 (activated or proenzyme). This observation is in accordance with recent data reported in an abstract presented at the last complement International Workshop in Newcastle (19). However, the lack of interaction of the proenzyme catalytic domain is somehow surprising because of the capacity of its active counterpart to cleave the N protein, which implies transient enzyme-substrate interaction. It may indicate that the MASP-2/N protein interaction involves additional N-terminal domains of the protease. It should be mentioned that co-authors of Gao et al. (15) reported previously that the SARS-CoV-1 N protein interacts with MAp19, a splicing product of MASP-2 encompassing the N-terminal CUB1-EGF segment of the protease and thus devoid of enzymatic activity (34). These former data are in apparent contradiction with the 2022 study where the authors found no interaction between SARS-N proteins and the N-terminal CUB1-EGF-CUB2 fragment of MASP-2 (15). We were not able to check the interaction of the N protein with this fragment by SPR because the purified recombinant fragment was not available to us. It may also be considered that the presence of the N-terminal domains of MASP-2 could stabilize the interaction of the N protein with the full-length protease.

The susceptibility of the N protein to proteolysis is not unexpected given its heterogeneous structure. Indeed, it comprises two globular RNA binding domains and three intrinsically disordered regions, located at the N- and C-terminal extremities and in the central region connecting the two structured domains (35). It has been found that the N protein undergoes proteolysis in the vicinity of the linker region, giving rise to several proteoforms (36). Notably, the flexible linker sequence contains several arginine residues corresponding to potential cleavage sites for trypsin-like proteases. MASP-2 has trypsin-like specificity and is known to cleave itself (autoactivation), complement C2 and C4 proteins, C1-Inhibitor, prothrombin (22, 37, 38) and more recently the HMGB1 alarmin (20). It is homologous to the C1s protease of the classical complement pathway and has been shown to have higher catalytic efficiency than C1s (39). It is therefore not surprising that C1s was also found to cleave the N protein, although less efficiently than MASP-2. A possible role for MASP-2 in the cleavage of the Spike protein of SARS-CoV-1 had also been suggested, based on comparison of data using recombinant MBL and serum-derived MBL (contaminated with MASPs) (18), which was not verified in the present study using recombinant SARS-CoV-2 Spike protein. It should be mentioned that the possible functional significance of N proteolysis by MASP-2 remains to be investigated. It may impact *in vitro* experiments including incubation steps with isolated recombinant proteins, which however do not reflect the complex physiological situation of infection. Importantly, serum MASP-2 is activated in the context of complexes with different pattern recognition proteins and we did not detect an effect of N protein on activation of the MBL-dependent lectin complement pathway. It has also been shown recently that the N protein synthesized during SARS-CoV-2 infection is released from cells and binds to both infected cells and bystander non-infected cells through association with heparan sulfate/heparin (40). In addition, cell-bound N protein was shown to interact with several chemokines and to modulate the host immune response.

In conclusion, we could not verify N protein-mediated activation of the lectin complement pathway (13) or potentiation of complement activation (15), in accordance with a recently published conference presentation abstract (19). We did not find essential differences between non-glycosylated N-Bact protein and the N-Expi glycosylated protein secreted by mammalian cells, in the context of our experiments. We also did not confirm the interaction of N protein with the catalytic domain of MASP-2, which had been reported in previous studies (13, 15). However, we could show that the N protein interacts with full-length MASP-2, but only with the protease in the proenzyme state. Finally, we provide evidence for the capacity of active MASP-2 to cleave the N protein, although the physiological significance of this cleavage in the serum of infected patients remains to be elucidated.

## Data availability statement

The raw data supporting the conclusions of this article will be made available by the authors, without undue reservation.

## Ethics statement

The studies involving humans were approved by Etablissement Français du Sang Auvergne Rhône Alpes 21-001. The studies were conducted in accordance with the local legislation and institutional requirements. The human samples used in this study were acquired from Etablissement Français du Sang Rhône-Alpes (agreement number EFS AURA 21-001 regarding its use in research). Written informed consent for participation was not required from the participants or the participants’ legal guardians/next of kin in accordance with the national legislation and institutional requirements.

## Author contributions

IB: Writing – review & editing, Conceptualization, Formal analysis, Investigation, Methodology. GD: Formal analysis, Investigation, Methodology, Writing – review & editing. VR: Investigation, Writing – review & editing. SG: Resources, Writing – review & editing. MBo: Resources, Writing – review & editing. J-BR: Investigation, Writing – review & editing. MT: Resources, Writing – review & editing. SDD: Formal analysis, Validation, Writing – review & editing. CD-P: Funding acquisition, Writing – review & editing. CG: Validation, Writing – review & editing. FF: Resources, Writing – review & editing. MBl: Resources, Writing – review & editing. PP: Funding acquisition, Writing – review & editing. NT: Conceptualization, Formal analysis, Funding acquisition, Investigation, Methodology, Project administration, Supervision, Validation, Writing – original draft, Writing – review & editing.
