# Supplementary material for: Revisiting the interaction between complement lectin pathway protease MASP-2 and SARS-CoV-2 nucleoprotein
Source: Front Immunol. 2024 Jun 7;15:1419165. doi: 10.3389/fimmu.2024.1419165 (PMC11190312; doi:10.3389/fimmu.2024.1419165)
Supplement: Supplementary file 1 [file DataSheet_1.pdf]

Supplementary Material

Revisiting the interaction between complement lectin pathway protease MASP-2 and SARS-CoV-2 nucleoprotein

Isabelle Bally, Guillaume Drumont, Véronique Rossi, Serafima Guseva, Maiia Botova, Jean-Baptiste Reiser, Michel Thépaut, Sebastian Dergan Dylon, Chantal Dumestre-Pérard, Christine Gaboriaud, Frank Fieschi, Martin Blackledge, Pascal Poignard, Nicole M. Thielens*

*** Correspondence:** Corresponding Author: Nicole.thielens@ibs.fr

Supplementary Figures

Figures S1 to S3

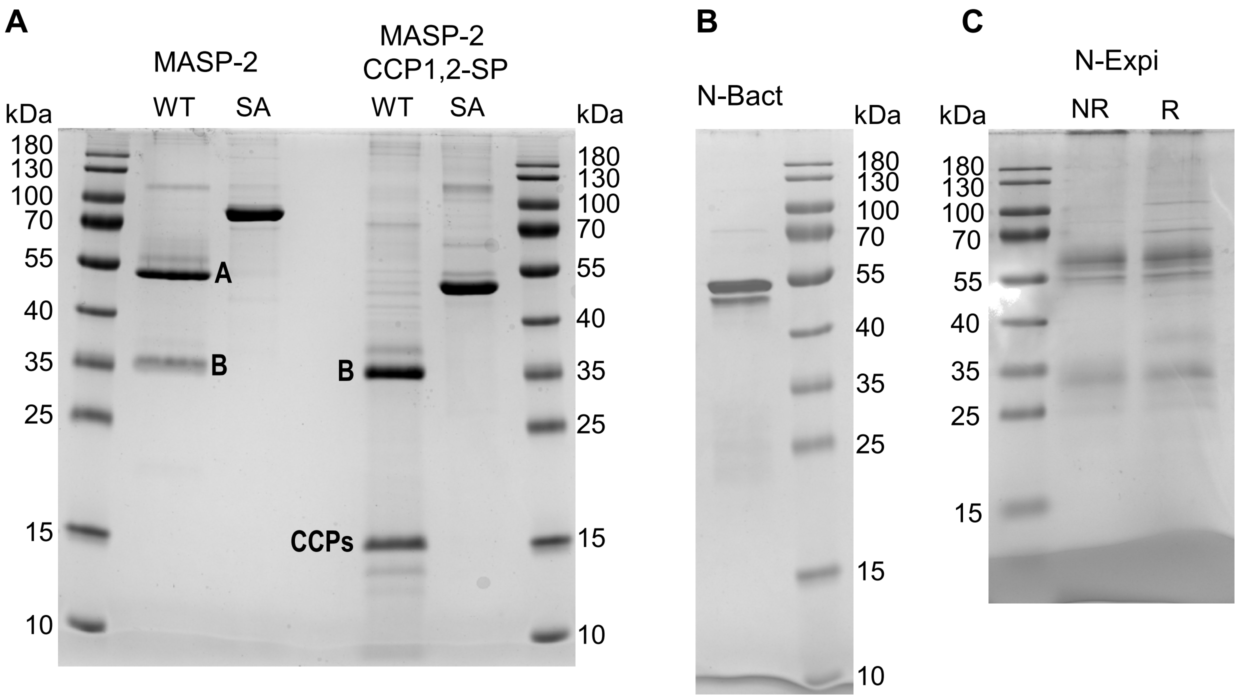

Supplementary Figure 1. SDS-PAGE analysis and Coomassie Blue staining of the human and viral purified recombinant proteins used in this study. **(A)** MASP-2 and its CCP1,2-SP catalytic domain, wild-type (WT) and S618A (SA) mutant (reducing conditions). The two chains of the activated enzymes are indicated whereas the SA mutants migrate as single bands. **(B)** N-Bact (reducing conditions). **(C)** N-Expi (non-reducing (NR) and reducing (R) conditions). Four μg of each protein were loaded on the gels (12.5% acrylamide). The molecular masses (kDa) of the markers are indicated.

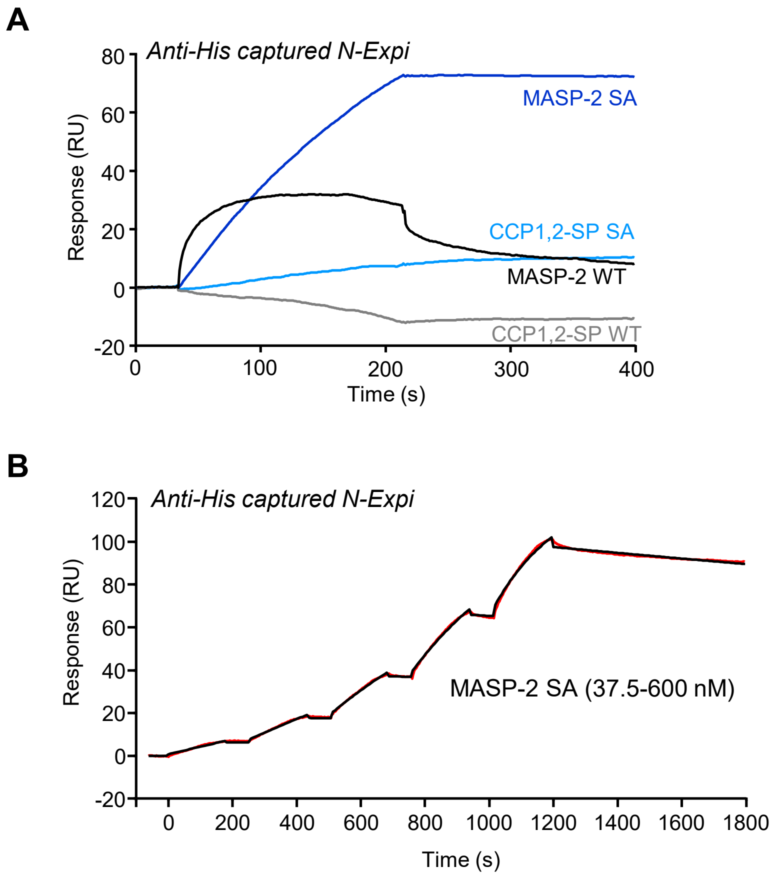

Supplementary Figure S2: SPR analyses of the interaction of MASP-2 proteins with N-Expi. **(A)** MASP-2 proteins (250 nM) were injected over 2,350 RU of N-Expi captured by covalently immobilized Penta-His antibody in TBS-Ca-T, pH 7.4 at a flow rate of 20 µl/min. The specific binding signal was obtained by subtracting the signal over the reference surface (flow cell with immobilized Penta-His antibody and without N captured). **(B)** MASP-2 S618A was serially diluted and injected at five increasing concentrations in single cycle kinetics mode over captured N-Expi (2,200 RU) in TBS-Ca-T at a flow rate of 20 µl/min. The fit (shown by a red line) was obtained by global fitting of the data to a Langmuir 1:1 binding model. The data shown are representative of 2 separate experiments on different surfaces.

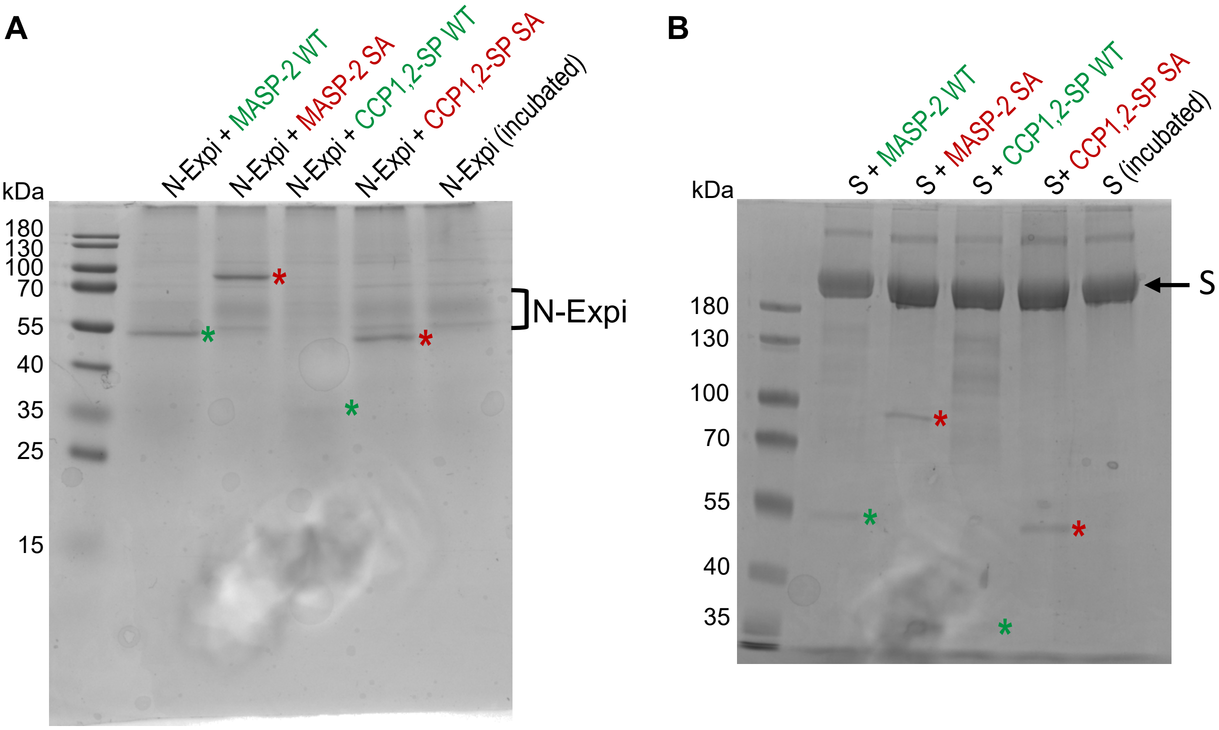

Supplementary Figure S3. MASP-2 proteolytic activity on **(A)** N-Expi and **(B)** Spike (S) proteins. N-Expi or S were incubated with 10% (molar ratio) wild-type activated MASP-2, its catalytic CCP1,2-SP fragment, their proenzyme S618A counterparts or TBS for 2 h at 37 °C. The digestion products were loaded on 12.5% **(A)** and 7.5% **(B)** acrylamide gels and SDS-PAGE analysis was performed under reducing conditions. The bands corresponding to the heavy chain of wild-type MASP-2 or its catalytic domain and to their single-chain proenzyme counterparts are indicated by green and red stars, respectively. The molecular masses (kDa) of the markers are indicated. Each gel shown is representative from two experiments.
